# Case Report: Successful intravascular ultrasound-guided percutaneous coronary intervention of a single coronary artery with chronic total occlusion and severe calcification using rotational atherectomy

**DOI:** 10.3389/fcvm.2026.1742040

**Published:** 2026-03-11

**Authors:** Kun Chen, Ning Guo

**Affiliations:** Changde Hospital, Xiangya School of Medicine, Central South University (The First People’s Hospital of Changde City), Changde, China

**Keywords:** chronic total occlusion, coronary calcification, intravascular ultrasound, percutaneous coronary intervention, rotational atherectomy, single coronary artery

## Abstract

An isolated single coronary artery (SCA) is a rare congenital anomaly, with an incidence of approximately 0.024%. The coexistence of chronic total occlusion (CTO) and severe calcification in a patient with SCA presents substantial procedural challenges during percutaneous coronary intervention (PCI). We report the case of a 52-year-old man with an anomalous right coronary artery (RCA) originating from the left main trunk (LMT), accompanied by proximal RCA CTO, a heavily calcified left anterior descending (LAD) artery, and subtotal occlusion of the left circumflex (LCX) artery. Coronary artery bypass grafting was declined, and a staged PCI was performed under intravascular ultrasound (IVUS) guidance. During the first procedure, stenting of the LCX artery and successful recanalization of the RCA CTO were performed. The second procedure included rotational atherectomy (RA) of the LAD artery, followed by stenting from the LMT to the LAD. Complete revascularization was achieved, and final IVUS imaging confirmed optimal stent expansion. This rare case illustrates that staged, IVUS-guided PCI can be performed safely and effectively in patients with an SCA and complex multivessel disease and that RA is a feasible and safe option for managing severe calcification in this unique anatomical setting.

## Introduction

An isolated single coronary artery (SCA) is a rare congenital anomaly with an angiographic prevalence of approximately 0.024% (1). While often asymptomatic, the specific variant in which a solitary ostium and trunk supply the entire myocardium confers a uniquely high-risk anatomical profile. The clinical implications of such anomalies remain poorly defined largely due to a reliance on retrospective data, making anatomy the primary guide for therapy. Critically, within this “single-conduit” system, any significant atherosclerotic disease poses an existential threat to global perfusion, predisposing patients to ischemia, arrhythmia, or sudden cardiac death (2, 3). In the already high-risk setting of an SCA, the coexistence of a chronic total occlusion (CTO) and severe calcification across multiple territories further compounds the technical and prognostic challenges of revascularization.

In cases of complex multivessel diseases involving an SCA, coronary artery bypass grafting (CABG) is the guideline-recommended strategy to ensure robust and durable myocardial protection (4, 5). Following a multidisciplinary heart team discussion, CABG was recommended for this patient. However, following the definitive refusal of surgery by the patient, percutaneous coronary intervention (PCI) was performed as the sole alternative. This decision mandated a PCI strategy designed to circumvent the cardinal risks of manipulating the solitary coronary ostium and trunk.

In this perilous anatomical setting, our procedural plan prioritized maximum safety and precision. We therefore adopted a staged, intravascular ultrasound (IVUS)-guided approach. Staging minimized per-procedural risk, while IVUS was integral for lesion assessment, device optimization, and result verification. To overcome the key barrier of severe circumferential calcification, we utilized rotational atherectomy (RA) for controlled plaque modification. This step was critical to ensure subsequent stent deliverability and to achieve optimal stent expansion (6–8).

## Case presentation

A 52-year-old man with a history of diabetes, dyslipidemia, and significant smoking status presented with typical anginal chest pain of 1 year duration. Electrocardiography was normal. Laboratory tests revealed normal levels of cardiac troponin, myocardial enzymes, NT-proBNP, and liver and renal function. The low-density lipoprotein cholesterol level was 2.33 mmol/L. Echocardiography showed mild tricuspid regurgitation with preserved left ventricular function.

**Figure 1:**
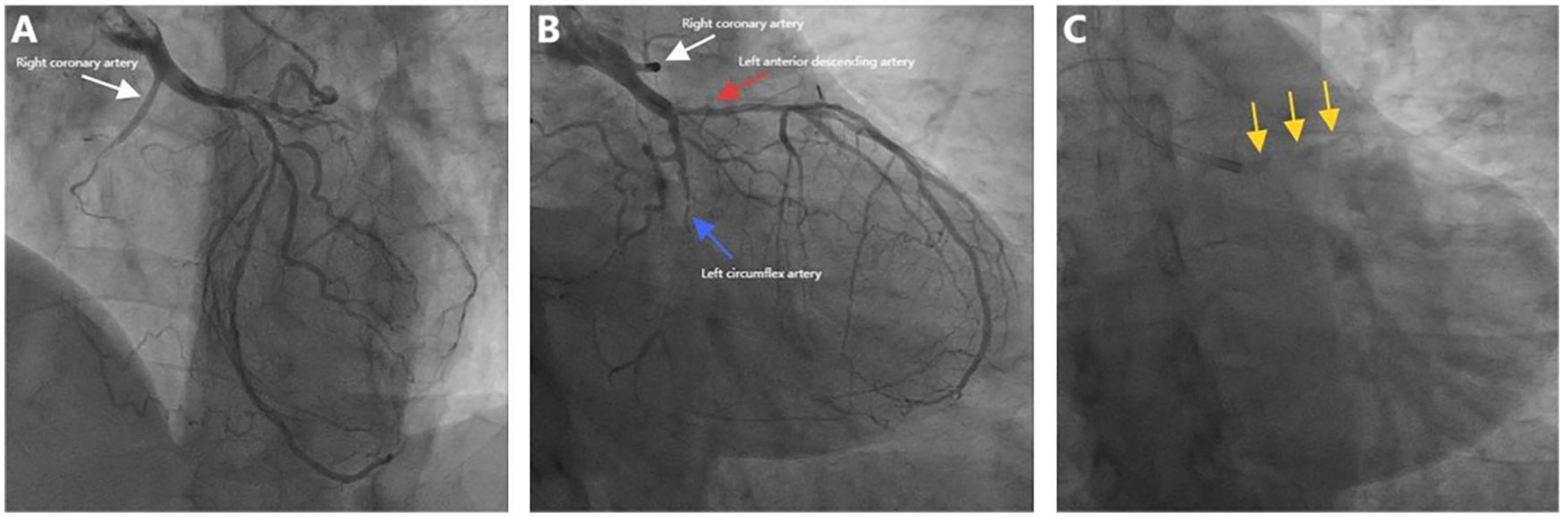
Baseline angiography images. **(A,B)** Anomalous RCA with proximal CTO (white arrow), subtotal LCX occlusion (blue arrow), and critical LAD stenosis (red arrow). **(C)** Severe LAD calcification (yellow arrows).

Coronary angiography revealed an anomalous right coronary artery (RCA) originating from the left main trunk (LMT) with CTO. In addition, there was severe calcification and critical stenosis of the left anterior descending (LAD) artery, as well as subtotal occlusion of the left circumflex (LCX) artery (Figures 1A–C, Supplementary Video S1). CABG was recommended but declined by the patient; therefore, a staged PCI was planned.

### Stage 1

**Figure 2:**
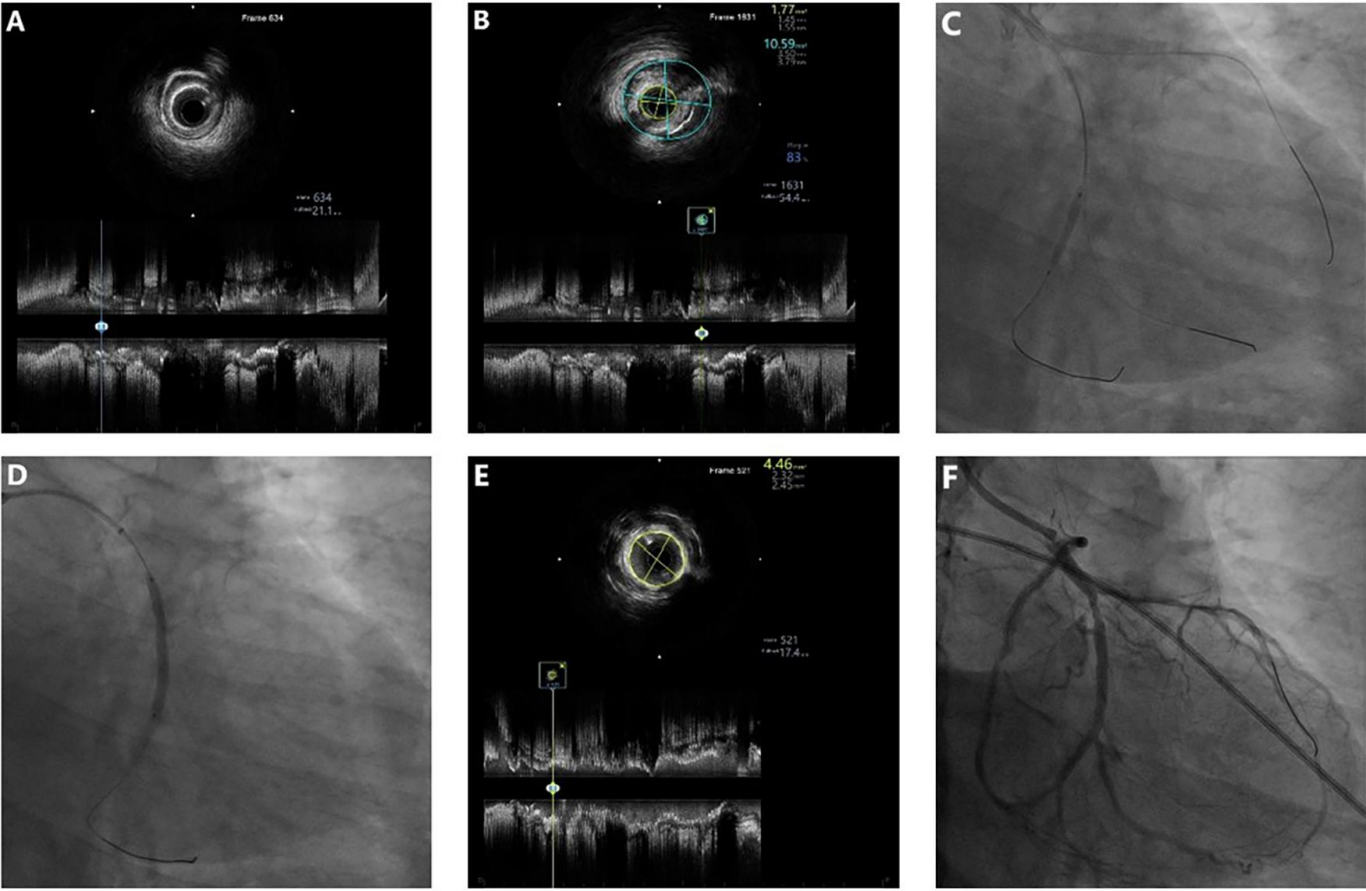
Baseline and post-PCI IVUS with stent implantation in the LCX. **(A,B)** Baseline IVUS showing fibrotic and calcified plaques (MLA 1.77 mm^2^, plaque burden 83%). **(C,D)** Sequential implantation of two DES (2.25 × 16 mm and 2.75 × 32 mm) in the LCX. **(E)** Post-PCI IVUS demonstrating MSA 4.46 mm^2^. **(F)** Final angiogram showing well-expanded LCX stents.

**Figure 3:**
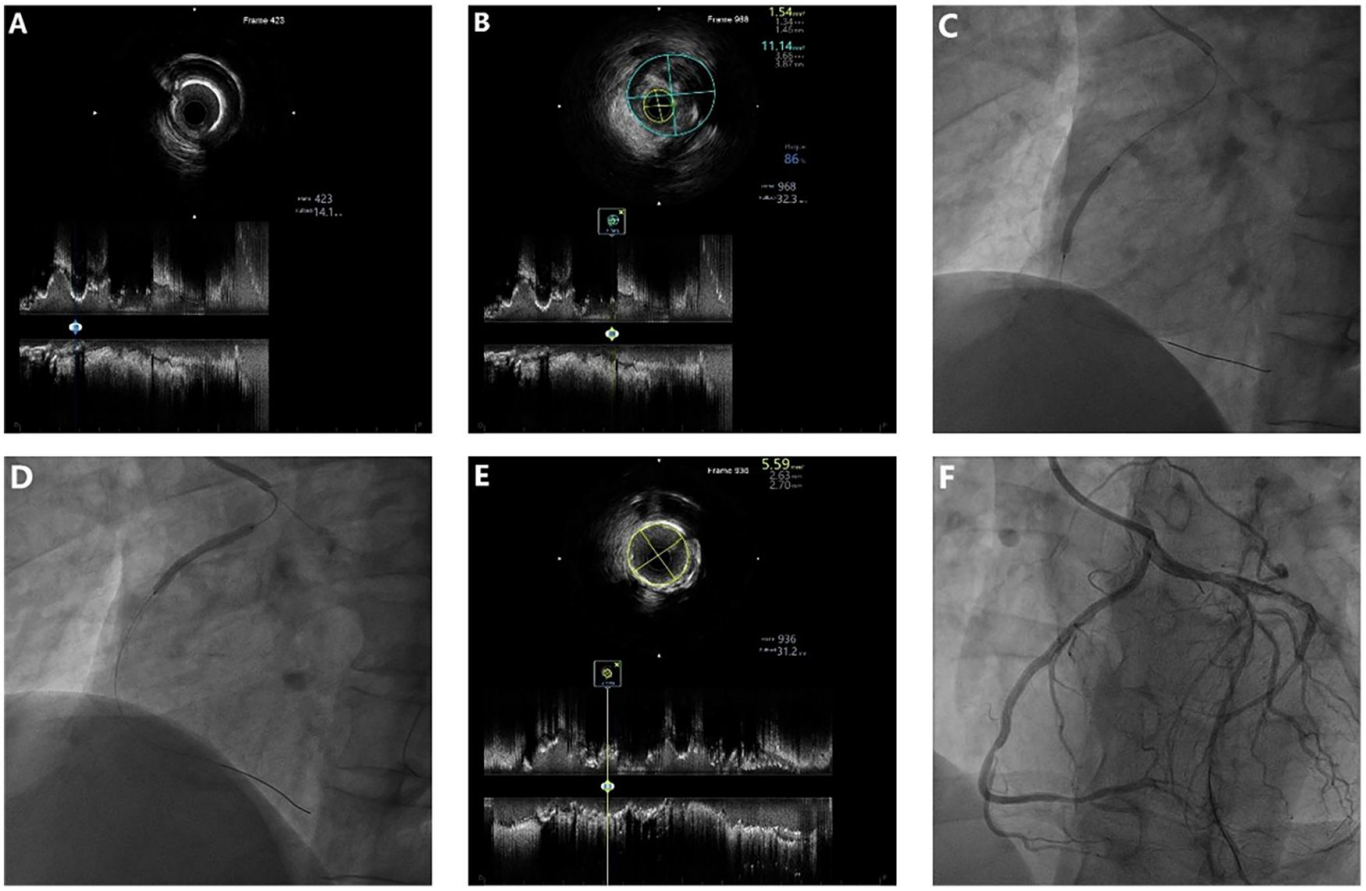
Baseline and post-PCI IVUS with stent implantation in the RCA. **(A,B)** Baseline IVUS showing fibrotic and calcified plaques (MLA 1.54 mm^2^, plaque burden 86%). **(C,D)** Sequential implantation of two DES (2.75 mm × 23 mm and 2.75 mm × 29 mm) in the RCA. **(E)** Post-PCI IVUS demonstrating an MSA of 5.59 mm^2^. **(F)** Final angiogram showing well-expanded RCA stents.

The initial procedure aimed to recanalize the occluded LCX and CTO of the anomalous RCA to improve overall coronary flow reserve. A 7F SAL1.0 guiding catheter was used to provide support via right radial access. The LCX occlusion was successfully crossed using a Fielder XT-R guidewire (Asahi Intecc, Nagoya, Japan) with microcatheter support. IVUS (Boston Scientific, Marlborough, MA, USA) revealed diffuse fibrocalcific plaques (Figures 2A,B). With support of a 4.3F × 150-cm guide extension catheter (YEAPRO), two overlapping SYNERGY^TM^ SHIELD everolimus**-**eluting stents (2.25 mm × 16 mm and 2.75 mm × 32 mm, Boston Scientific) were deployed (Figures 2C,D), followed by sequential postdilation. IVUS confirmed adequate stent expansion, with a minimal stent area (MSA) of 4.46 mm^2^ (Figure 2E), resulting in excellent angiographic results (Figure 2F). Subsequently, focusing on the RCA CTO, a Gaia Second (Asahi Intecc) guidewire was advanced through a microcatheter and successfully crossed the occlusion. IVUS verified true lumen position and demonstrated similar plaque morphology (Figures 3A,B). Two sirolimus-eluting stents (Firebird2, 2.75 mm × 23 mm and 2.75 mm × 29 mm; Microport Medical, Shanghai, China) were then implanted (Figures 3C,D), followed by postdilation. Final IVUS confirmed good stent expansion (MSA 5.59 mm^2^) (Figure 3E), and an excellent angiographic result was achieved (Figure 3F).

The interventional procedures for the LCX and RCA are presented in Supplementary Videos S2, S3.

### Stage 2

**Figure 4:**
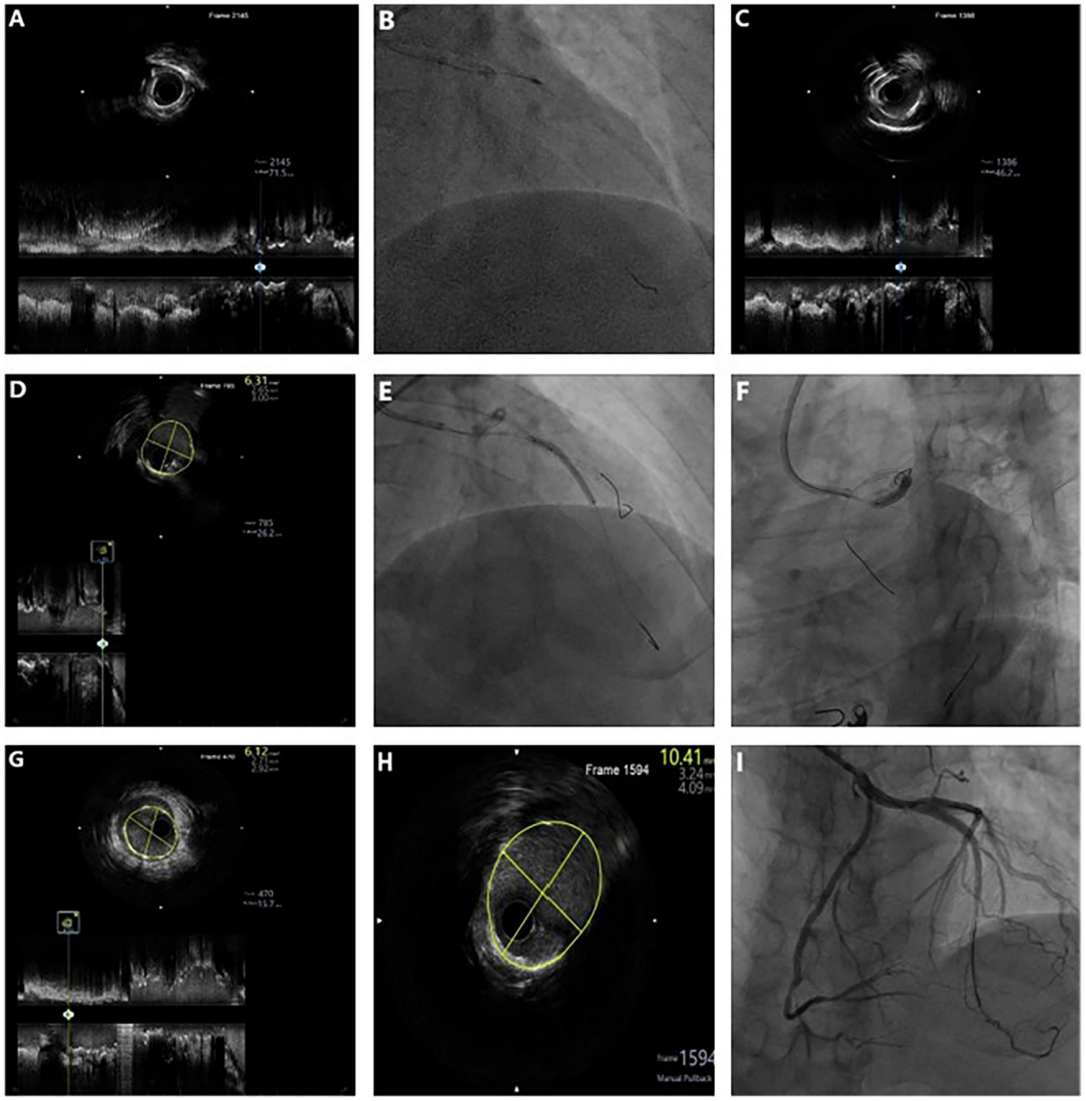
Stepwise IVUS- and RA-guided PCI showing severe calcification and optimal stent expansion in the LAD-LMT. **(A)** Baseline IVUS showing severe circumferential calcification in the LAD-LMT. **(B)** RA with a 1.5-mm burr in the LAD-LMT. **(C)** Post-RA demonstrating multiple reflection artifacts. **(D)** IVUS of the LCX ostium revealing a lipid plaque with an MLA of 6.31 mm^2^. **(E,F)** Sequential implantation of two DES (2.75 × 28 mm and 3.0 × 20 mm) from the LAD to the LMT. **(G,H)** Poststenting IVUS confirming adequate stent expansion (MSA: 6.12 mm^2^ in the LAD and 10.41 mm^2^ in the LMT). **(I)** Final angiogram showing well-expanded LAD-LM stents.

The second stage addressed the paramount challenge of severe calcification of the proximal LAD involving the LMT. Using a 7F EBU3.75 guiding catheter via radial access, baseline IVUS confirmed near-circumferential calcification (Figure 4A). To protect the major side branches, the first diagonal branch and a major septal branch were predilated with a 1.5 mm × 15 mm semi-compliant balloon. RA was then performed using a 1.5-mm burr (Boston Scientific) at 150,000 rpm (Figure 4B, Supplementary Video S4). Post-RA IVUS demonstrated effective plaque modification, characterized by multiple reverberation artifacts (Figures 4C). IVUS assessment of the LCX ostium revealed an adequate MSA of 6.31 mm^2^, obviating the need for additional intervention (Figure 4D). After confirming full lesion expansion with a non-compliant balloon, two overlapping Promus PREMIER™ everolimus-eluting stents (2.75 mm × 28 mm and 3.0 mm × 24 mm, Boston Scientific) were deployed from the proximal LAD to the LMT (Figures 4E,F). Final optimization with sequential balloon dilations yielded optimal stent expansion on IVUS (LMT MSA, 10.41 mm^2^; LAD MSA, 6.12 mm^2^) without complications (Figures 4G,H), achieving complete revascularization with excellent angiographic results (Figure 4I).

The interventional procedures for LAD-LMT are presented in Supplementary Video S5.

## Postprocedural course and follow-up

The patient tolerated both procedures well, with no peri-procedural complications such as coronary dissection, perforation, or slow flow. He was discharged on dual antiplatelet therapy, a statin, and other guideline-directed secondary prevention medications and was advised to implement healthy lifestyle modifications. At 10-month follow-up, he remained asymptomatic, with normal exercise tolerance and no evidence of myocardial ischemia on stress testing.

## Discussion

### Anatomical features and risk assessment of SCA

Coronary artery anomalies (CAAs) are classified based on abnormalities in their origin, course, and termination (3). In this case, angiography revealed an isolated SCA, with an anomalous right coronary artery originating from the LMT. Although coronary computed tomography angiography was not performed to definitively exclude an interarterial course (a feature of the higher-risk L-II type), the angiographic findings were most consistent with a Lipton L-IIA pattern (1, 9, 10). Regardless of the subtype, the fundamental risk stems from the “single-conduit” anatomy. Consequently, any complication or suboptimal treatment of a proximal lesion within this sole conduit could result in catastrophic, extensive myocardial ischemia (11, 12). The presence of complex, calcified multivessel disease—including an RCA CTO and a critically stenotic LAD—within this SCA mandated a revascularization strategy that prioritized absolute safety, precision, and durability, forming the basis for our staged, IVUS-guided PCI approach with adjunctive RA (13).

### Technical challenges and strategic considerations of PCI in SCA

PCI in the setting of an SCA carries a risk profile that transcends that of standard complex PCI, as the entire myocardium depends on a solitary ostium and proximal conduit. Procedural complications, such as ostial dissection or prolonged ischemia, therefore, increase the risk of precipitating global myocardial infarction (14–16). This shifts the overarching principle from technical success to absolute safety and risk mitigation, a nuance not captured by conventional risk scores (e.g., the SYNTAX score) (5, 17, 18). Although CABG is preferred because it establishes alternative conduits (4, 19), PCI becomes a necessary, yet exceptionally delicate, alternative when surgery is declined, relying heavily on meticulous planning and intravascular imaging guidance.

Our staged, imaging-guided strategy was meticulously designed to mitigate these specific SCA-related risks as follows:

1. Mitigating hemodynamic collapse: A staged strategy was employed to minimize the ischemic time and contrast load per procedure. Although preemptive circulatory support devices (e.g., intra-aortic balloon pump, Impella) (20, 21) were available for potential intraprocedural instability, their use was ultimately not required because of the patient's preserved left ventricular function and the inherent risk mitigation provided by the staged approach.
2. Ensuring absolute procedural control and safety: Aggressive catheter support (7-Fr guides with guide extension) was non-negotiable to prevent ostial trauma and ensure single-pass device delivery. A highly trackable microcatheter was critical for navigating the acute angulation of the anomalous RCA while avoiding traumatic manipulation (22).
3. Prioritizing physiological and anatomical optimization: The sequence was physiologically rational. Stage 1 (LCX and RCA) first restorated blood flow to a large territory, improving ischemic reserve before tackling the highest-risk lesion (calcified LAD) in Stage 2.
4. Bailout planning and precision execution: Universal IVUS use was a central component of our safety strategy, providing definitive confirmation of guidewire position, adequate calcium modification, and optimal stent results at each step, thereby preventing geographic miss or suboptimal stent expansion that could trigger vessel closure.

### Role of IVUS in procedural optimization of Complex PCI

Given these imperatives, IVUS transitioned the procedure from angiographic estimation to a quantitative vessel wall-based decision-making approach. It played a pivotal role at every phase: confirming true lumen position after guidewire crossing, characterizing plaque morphology to inform device selection, guiding optimal balloon and stent sizing, verifying adequate calcium modification after RA, and ultimately ensuring optimal stent expansion and apposition while excluding complications (23, 24). This comprehensive imaging guidance is indispensable for both procedural safety and long-term outcome optimization.

### Rotational atherectomy for severe calcification in a single coronary artery

Severe coronary calcification is a paramount predictor of stent underexpansion, target lesion failure, and subsequent major adverse cardiovascular events (MACEs) (22, 25–27). In the setting of a SCA, achieving optimal stent expansion is critical to prevent future catastrophic ischemia events. Therefore, the selection of a plaque modification technique was guided by the need to maximize safety and controllability while securing sufficient luminal gain within this sole, irreplaceable conduit.

The contemporary armamentarium for modifying severely calcified lesions comprises three principal categories (22, 28). The rationale for selecting RA over these options is as follows:

1. Balloon-based techniques: Modalities such as cutting or scoring balloons lack true ablative capacity and often fail to adequately modify deep calcium or provide sufficient luminal gain for stent expansion.
2. Intravascular lithotripsy (IVL): Although IVL offers an excellent safety profile in standard anatomy (23, 24), its application in the setting of an SCA necessitates a heightened risk assessment. A primary concern is the transient coronary occlusion during balloon inflation, which poses a significant risk of extensive myocardial ischemia in this sole conduit. Furthermore, although IVL fractures calcium, it does not actively debulk plaque to facilitate luminal gain.
3. Alternative atherectomy devices: Orbital atherectomy and excimer laser coronary atherectomy (ELCA) were not available at our center. Available literature indicates that laser atherectomy has limited efficacy against severe, circumferential calcification and a less predictable safety profile, making it a suboptimal choice for this high-risk scenario (29).

#### Selection of rotational atherectomy

Among the available options, RA was selected as the optimal strategy. By adhering to a pecking-motion technique with a small burr at a speed deliberately set at the lower end of the recommended spectrum, we achieved precise, tactile-guided plaque modification while minimizing vessel trauma—a critical consideration for preserving the solitary SCA conduit (22, 30–33).

In this case, postatherectomy IVUS confirmed effective plaque modification, and subsequent stent optimization achieved satisfactory expansion without complications, supporting the feasibility of a carefully executed RA procedure in this high-risk anatomical variant.

## Conclusion

This case describes a successful staged, IVUS-guided PCI strategy incorporating RA for the treatment of complex, calcified multivessel disease in the setting of an SCA. To our knowledge, a procedural account of RA use in this specific adult anatomical variant has not been previously reported. Our experience underscores that, in such ultrahigh-risk anatomy, success hinges on a comprehensive safety-first protocol—integrating procedural staging, universal imaging guidance, and controlled lesion modification—rather than on any single device. Therefore, this report is not intended to claim primacy but rather to provide a validated technical blueprint and to highlight the critical role of tailored, imaging-based planning in managing similar scenarios in contemporary interventional practice.

## Data Availability

The original contributions presented in the study are included in the article/Supplementary Material, further inquiries can be directed to the corresponding author.

## References

[1] Lipton MJ Barry WH Obrez I Silverman JF Wexler L. Isolated single coronary artery: diagnosis, angiographic classification, and clinical significance. Radiology. (1979) 130(1):39–47. 10.1148/130.1.39 758666

[2] Angelini P. Coronary artery anomalies: an entity in search of an identity. Circulation. (2007) 115(10):1296–305. 10.1161/CIRCULATIONAHA.106.618082 17353457

[3] Gentile F Castiglione V De Caterina R. Coronary artery anomalies. Circulation. (2021) 144(12):983–96. 10.1161/CIRCULATIONAHA.121.055347 34543069

[4] Stout KK Daniels CJ Aboulhosn JA Bozkurt B Broberg CS Colman JM 2018 AHA/ACC guideline for the management of adults with congenital heart disease: a report of the American College of Cardiology/American Heart Association task force on clinical practice guidelines. Circulation. (2019) 139(14):e698–800. 10.1161/CIR.0000000000000603 30586767

[5] Thuijs DJFM Kappetein AP Serruys PW Mohr FW Morice MC Mack MJ Percutaneous coronary intervention versus coronary artery bypass grafting in patients with three-vessel or left main coronary artery disease: 10-year follow-up of the multicentre randomised controlled SYNTAX trial. Lancet. (2019) 394(10206):1325–34. 10.1016/S0140-6736(19)31997-X 31488373

[6] Zhang J Gao X Kan J Ge Z Han L Lu S Intravascular ultrasound versus angiography-guided drug-eluting stent implantation. J Am Coll Cardiol. (2018) 72(24):3126–37. 10.1016/j.jacc.2018.09.013 30261237

[7] Rheude T Fitzgerald S Allali A Mashayekhi K Gori T Cuculi F Rotational atherectomy or balloon-based techniques to prepare severely calcified coronary lesions. JACC Cardiovasc Interv. (2022) 15(18):1864–74. 10.1016/j.jcin.2022.07.034 36137691

[8] Gao XF Ge Z Kong XQ Chen X Han L Qian XS Intravascular ultrasound vs angiography-guided drug-coated balloon angioplasty: the ULTIMATE Ⅲ trial. JACC Cardiovasc Interv. (2024) 17(13):1519–28. 10.1016/j.jcin.2024.04.014 38842991

[9] Yamanaka O Hobbs RE. Coronary artery anomalies in 126,595 patients undergoing coronary arteriography. Cathet Cardiovasc Diagn. (1990) 21(1):28–40. 10.1002/ccd.1810210110 2208265

[10] Said SA de Voogt WG Bulut S Han J Polak P Nijhuis RL Coronary artery disease in congenital single coronary artery in adults: a Dutch case series. World J Cardiol. (2014) 6(4):196–204. 10.4330/wjc.v6.i4.196 24772259 PMC3999339

[11] Chaikriangkrai K Kassi M Polsani V Chang SM. Case report: single coronary artery with ischemia and sudden cardiac arrest. Methodist DeBakey Cardiovasc J. (2014) 10(2):121–3. 10.14797/mdcj-10-2-121 25114765 PMC4117331

[12] Neiva J Passos Silva M Pires-Morais G Dias A Ponte M Caeiro D Right single coronary artery as an incidental finding in Takotsubo syndrome and acute heart failure: case report and review of the literature. Rev Port Cardiol. (2019) 38(3):215–23. 10.1016/j.repc.2018.06.013 31014998

[13] Musiani A Cernigliaro C Sansa M Maselli D De Gasperis C. Left main coronary artery atresia: literature review and therapeutical considerations. Eur J Cardio-thorac Surg. (1997) 11(3):505–14. 10.1016/S1010-7940(96)01121-9 9105816

[14] Çitaku H Krasniqi X. Catheter left main dissection bailout treatment with stenting. Open Access Maced J Med Sci. (2019) 7(7):1180–3. 10.3889/oamjms.2019.253 31049104 PMC6490492

[15] Acar B Arıkan AA Talas Z Celikyurt U Kanko M. Stent implantation may fail sometimes in coronary complications: extension of an iatrogenic left main coronary artery hematoma. J Tehran Heart Cent. (2022) 17(4):249–51. 10.18502/jthc.v17i4.11615 37143753 PMC10154107

[16] Morosato M Gaspardone C Romagnolo D Pagnesi M Baldetti L Dormio S Left main spontaneous coronary artery dissection: clinical features, management, and outcomes. JACC Cardiovasc Interv. (2025) 18(8):975–83. 10.1016/j.jcin.2025.01.427 40208153 PMC12290918

[17] Takahashi K Serruys PW Fuster V Farkouh ME Spertus JA Cohen DJ Redevelopment and validation of the SYNTAX score II to individualise decision making between percutaneous and surgical revascularisation in patients with complex coronary artery disease: secondary analysis of the multicentre randomised controlled SYNTAXES trial with external cohort validation. Lancet (Lond Engl). (2020) 396(10260):1399–412. 10.1016/S0140-6736(20)32114-0 33038944

[18] Sianos G Morel MA Kappetein AP Morice MC Colombo A Dawkins K The SYNTAX score: an angiographic tool grading the complexity of coronary artery disease. EuroIntervention. (2025) 1(2):219–27. https://eurointervention.pcronline.com/article/the-syntax-score-an-angiographic-tool-grading-the-complexity-of-coronary-artery-disease 19758907

[19] Baumgartner H De Backer J Babu-Narayan SV Budts W Chessa M Diller GP 2020 ESC guidelines for the management of adult congenital heart disease. Eur Heart J. (2021) 42(6):563–645. 10.1093/eurheartj/ehaa554 32860028

[20] Ameloot K Bastos MB Daemen J Schreuder J Boersma E Zijlstra F New-generation mechanical circulatory support during high-risk PCI: a cross-sectional analysis. EuroIntervention. (2019) 15(5):427–33. 10.4244/EIJ-D-18-01126 30741638

[21] Thiele H Hassager C. Cardiogenic shock. N Engl J Med. (2026) 394(1):62–77. 10.1056/NEJMra2312086 41467651

[22] Barbato E Gallinoro E Abdel-Wahab M Andreini D Carrié D Di Mario C Management strategies for heavily calcified coronary stenoses: an EAPCI clinical consensus statement in collaboration with the EURO4C-PCR group. Eur Heart J. (2023) 44(41):4340–56. 10.1093/eurheartj/ehad342 37208199

[23] Mintz GS Matsumura M Ali Z Maehara A. Clinical utility of intravascular imaging: past, present, and future. JACC Cardiovasc Imaging. (2022) 15(10):1799–820. 10.1016/j.jcmg.2022.04.026 36202460

[24] Giacoppo D Laudani C Occhipinti G Spagnolo M Greco A Rochira C Coronary angiography, intravascular ultrasound, and optical coherence tomography for guiding of percutaneous coronary intervention: a systematic review and network meta-analysis. Circulation. (2024) 149(14):1065–86. 10.1161/CIRCULATIONAHA.123.067583 38344859 PMC10980178

[25] Copeland-Halperin RS Baber U Aquino M Rajamanickam A Roy S Hasan C Prevalence, correlates, and impact of coronary calcification on adverse events following PCI with newer-generation DES: findings from a large multiethnic registry. Catheter Cardiovasc Interv. (2018) 91(5):859–66. 10.1002/ccd.27204 28722295

[26] Guedeney P Claessen BE Mehran R Mintz GS Liu M Sorrentino S Coronary calcification and long-term outcomes according to drug-eluting stent generation. JACC Cardiovasc Interv. (2020) 13(12):1417–28. 10.1016/j.jcin.2020.03.053 32553329

[27] Rheude T Koch T Joner M Lenz T Xhepa E Wiebe J Ten-year clinical outcomes of drug-eluting stents with different polymer coating strategies by degree of coronary calcification: a pooled analysis of the ISAR-TEST 4 and 5 randomised trials. Eurointervention. (2023) 18(14):1188–96. 10.4244/EIJ-D-22-00781 36453826 PMC9936252

[28] De Maria GL Scarsini R Banning AP. Management of calcific coronary artery lesions. JACC Cardiovasc Interv. (2019) 12(15):1465–78. 10.1016/j.jcin.2019.03.038 31395217

[29] Golino L Caiazzo G Calabrò P Colombo A Contarini M Fedele F Excimer laser technology in percutaneous coronary interventions: cardiovascular laser society’s position paper. Int J Cardiol. (2022) 350:19–26. 10.1016/j.ijcard.2021.12.054 34995700

[30] Abdel-Wahab M Toelg R Byrne RA Geist V El-Mawardy M Allali A High-speed rotational atherectomy versus modified balloons prior to drug-eluting stent implantation in severely calcified coronary lesions. Circ Cardiovasc Interv. (2018) 11(10):e007415. 10.1161/CIRCINTERVENTIONS.118.007415 30354632

[31] Jurado-Román A Gómez-Menchero A Rivero-Santana B Amat-Santos IJ Jiménez-Valero S Caballero-Borrego J Rotational atherectomy, lithotripsy, or laser for calcified coronary stenosis: the ROLLER COASTR-EPIC22 trial. JACC Cardiovasc Interv. (2025) 18(5):606–18. 10.1016/j.jcin.2024.11.012 39918495

[32] Yabushita H Takagi K Tahara S Fujino Y Warisawa T Kawamoto H Impact of rotational atherectomy on heavily calcified, unprotected left main disease. Circ J. (2014) 78(8):1867–72. 10.1253/circj.CJ-13-1426 24920410

[33] Yoshida R Ishii H Morishima I Tanaka A Tsuda T Takagi K Ablation effect of additional low-speed rotational atherectomy following high-speed rotational atherectomy: low-speed RA following high-speed RA. AsiaIntervention. (2020) 6(1):52–5. 10.4244/AIJ-D-19-00022 34912985 PMC8525725

